# Should the early surgery threshold be moved to 72 h in over-85 patients with hip fracture? A single-center retrospective evaluation on 941 patients

**DOI:** 10.1007/s00402-022-04509-y

**Published:** 2022-07-05

**Authors:** Alessandro De Luca, Luigi Murena, Michela Zanetti, Paolo De Colle, Chiara Ratti, Gianluca Canton

**Affiliations:** 1 Orthopedics and Traumatology Unit, Department of Medical Surgical and Health Sciences, Cattinara Hospital-ASUGI, Trieste University, Strada di Fiume 447, 34149 Trieste, Italy; 2 Department of Medical, Surgical and Health Sciences, Geriatric Unit ASUGI, University of Trieste, Trieste, Italy; 3 Department of Medicine ASUGI, Surgery, and Health Sciences, University of Trieste, Trieste, Italy

**Keywords:** Early surgery, Elderly, Fragility fractures, Hip fractures, Mortality

## Abstract

**Aim:**

Aim of the study was to assess whether early surgery and other clinical and orthogeriatric parameters could affect mortality rate in hip fracture patients aged > 85.

**Materials and methods:**

Data regarding a 42-month period were retrospectively obtained from the institutional medical records and registry data. Gender, age, fracture pattern, surgical technique, type of anesthesia, timing of surgical intervention (within 24, 48 or 72 h from admission), days of hospitalization, mortality rate divided in intra-hospital, at 30 days and at 1 year were collected for the whole population. Some additional data were collected for an orthogeriatric subgroup.

**Results:**

941 patients were considered, with a mean age of 89 years. Surgery was performed within 24, 48 and 72 h in 24.4%, 54.5% and 66.1% of cases, respectively. Intra-hospital mortality rate resulted to be 3.4%, while mortality at 30 days and 1 year resulted to be 4.5% and 31%, respectively. Early surgery within 48 and 72 h were significantly associated with a lower intra-hospital and 30-day mortality rate. In the orthogeriatric subgroup (394 patients), a significant association with a higher mortality rate was found for general anesthesia, number of comorbidities, ADL (Activities of Daily Living) < 3, transfer to other departments.

**Conclusions:**

In over-85 hip fracture patients, the threshold for early surgery might be moved to 72 h to allow patients pre-operative stabilization and medical optimization as intra-hospital and 30-day mortality rates remain significantly lower. Advanced age, male sex, number of comorbidities, pre-operative dependency in ADL, general anesthesia, length of hospitalization and transfer to other departments were significantly related to mortality rate.

## Introduction

Proximal femur fracture (PFF) is one of the most frequent fractures in the elderly population, with severe clinical and socio-economic implications.

Due to the localization of the fracture, and in order to prevent complications related to prolonged immobilization, surgery is performed in more than 90% of cases [1, 2].

Although only 2.5% of the Italian population exceeds the age of 85 years, over 40–50% of patients hospitalized for proximal femur fractures are included in this age group, defined as “oldest old,” according to the higher prevalence of osteoporosis [3–5]. Nevertheless, there are few studies that deal purely with the geriatric population, as most papers about PFF treatment consider patients aged over 65 years. Intra-hospital mortality appears to be around 5% [1, 6], while mortality at 30 days is reported to be between 5 and 10% [4, 5, 7, 8], with peaks up to 15% in the over 80-years old [9, 10]. Mortality 1 year after surgery stands around 20 to 30% in most studies [1, 11–18].

The timing of surgery still remains a high debated issue. Some literature reports state that surgical intervention must be carried out within 24 h of admission [19–30]. Many other authors report a reduced mortality in case of early intervention within 48 h, both in the short and in the long term [4, 19, 24, 25, 31–34]. The advantages of early surgery would reside in a better functional outcome, a reduced hospitalization, fewer complications, reduced pain and a reduced risk of death [28, 31, 33]. However, there is a part of the literature that does not find benefits in terms of mortality from early intervention [7, 34–37] These authors claim that unstable patients must be stabilized first, even at the expense of operative timing [6, 7].

Beside literature conflicting results, the value of early surgery is generally accepted. Thus, surgery realized within 48 h after PFF is one of the mostly used indicators of healthcare quality [2].

Aim of the study was to assess whether early surgery (within 24, 48 or 72 h) for PFF in patients aged > 85 affects intra-hospital, 30-days and 1-year mortality rate, in order to define the more accurate early surgery time threshold for these patients. Secondary aim was to assess which other variables, including clinical and orthogeriatric parameters, could influence the mortality rate in this age group.

## Materials and methods

All patients surgically treated at a single trauma center for PFF between January 1, 2014, and June 30, 2017, were initially considered.

Exclusion criteria were: patients aged under 85, PFF in the antecedent 2 years, patients in critical conditions who were treated in intensive or semi-intensive care unit before surgery, polytrauma, patients deceased within 48 h from admission without undergoing surgery, oncologic patients with metastatic disease, pathologic fractures, patients treated conservatively, simultaneous bilateral fracture, patients with incomplete/lacking documentation.

Data were retrospectively obtained from the institutional medical records and registry data, and any death was taken from nationwide database until June 30, 2018.

The records collected for the whole population were: gender, age, fracture pattern (medial/intracapsular vs lateral/extracapsular), surgical technique (partial hip replacement vs osteosynthesis), type of anesthesia, timing of surgical intervention (within 24, 48 or 72 h from admission), days of hospitalization (from surgery to discharge and total), mortality rate divided in intra-hospital, at 30 days and at 1 year.

Since a PFF registry was introduced from January 2016 through the collaboration with the orthogeriatric service, some addictive data could be collected for patients admitted form January 2016 to June 2017 (orthogeriatric subgroup). These data included: number of drugs per day; type of drugs, divided in six subcategories (anticoagulant, antiaggregant, antidiabetics, drugs acting on the CNS, corticosteroids/immune-suppressive drugs and antiresorptive drugs); comorbidities, divided in six subcategories (non-complicated hypertension, cardiovascular diseases excluding non-complicated hypertension, respiratory and neurologic diseases excluding dementia, metabolic diseases including diabetes and renal failure); ADL (Activities of Daily Living) score pre-admission, dividing patients into dependent for ADL (score 0–3) and independent for ADL (score 4–6); post-operative delirium; discharge destination (home, retirement home, healthcare residences, rehabilitation facilities and other intra-hospital departments).

The statistical analysis was performed using the SPSS Statistics software. Mean and standard deviation were calculated for quantitative variables.

Correlation between timing of surgery and mortality at the three set end-points was calculated with the chi-square test on the whole population. A univariate binary logistic regression model was used to identify the correlation between the collected parameters and mortality at the three set end-points, setting mortality as dependent variable. Statistical significance was set for a *p* value < 0.05. The odds ratio was calculated in order to define the relative risk of mortality for each significant independent covariate.

Samples too small for meaningful statistical analysis were discarded.

## Results

The population considered for the study counted 2342 patients admitted with a diagnosis of PFF.

The characteristics of our sample are summarized in Table 1.

**Table 1:** Descriptive data of the whole population studied

	Parameter	*N*°	%
Total		941	100
Gender	F	772	82
	M	169	18
Fracture pattern	Medial	442	47
	Lateral	499	53
Surgical technique	Osteosynthesis	512	54.4
	Hip replacement	429	45.6
Type of anesthesia	General	71	7.5
	Spinal	850	90.3
	Unknown	20	2.1
Timing of surgical intervention	< 24 h	230	24.4
	< 48 h	513	54.5
	< 72 h	622	66.1
Mortality rate	Intra-hospital	32	3.4
	30 days	42	4.5
	1 year	292	31

	Mean	SD	Range
Age (years)	89.86	3.5	85–107
Total hospital stay (days)	17.64	10.257	3–153
Post-op hospital stay (days)	14.2	9.32	0–147

Intra-hospital mortality rate of the whole population resulted to be 3.4%, while mortality at 30 days and 1 year resulted to be 4.5% and 31%, respectively.

The orthogeriatric subgroup counted 394 patients. Demographic data and additional information as type and number of drugs, comorbidities and discharge destination are resumed in Table 2.

**Table 2:** Descriptive data of the orthogeriatric subgroup

	Parameter	*N*°	%
Total		394	
Gender	F	311	78.9
	M	83	21.1
Fracture pattern	Medial	178	45.2
	Lateral	216	54.8
Surgical technique	Fixation	223	56.6
	Hip replacement	171	43.4
Type of anesthesia	General	40	10.2
	Spinal	341	86.5
	Unknown	20	3.3
Timing of surgical intervention	< 24 h	168	42.6
	< 48 h	318	80.7
	< 72 h	345	87.5
Mortality rate	Intra-hospital	6	1.5
	30 days	15	3.8
	1 year	127	32.2
Number of drugs per day	0	23	5.8
	1	27	6.9
	2	36	9.1
	3	51	12.9
	4	62	15.7
	5	56	14.2
	6	43	10.9
	7	41	10.4
	8	28	7.1
	9	8	2
	10	10	2.5
	11	5	1.3
	12	2	0.5
	13	2	0.5
	3–7	253	64.2
	> 10	19	4.8
Type of drug	Anticoagulant	35	8.9
	Antiaggregant	161	40.9
	Antidiabetics	62	15.7
	Acting on the CNS	197	50
	Corticosteroids/ immune-suppressive	18	4.6
	Antiresorptive	77	19.5
Comorbidities	0	80	20.3
	1	104	26.4
	2	111	28.2
	3	66	16.8
	4	29	7.4
	5	4	1
Comorbidities subcategories	Non-complicated hypertension	252	64
	Cardiovascular (excluding non-complicated hypertension)	173	43.9
	Respiratory diseases	45	11.4
	Neurologic diseases (excluding dementia)	42	10.7
	Metabolic diseases including diabetes	63	16
	Renal failure	84	21.3
Post-operative delirium		57	14.5
ADL (activities of daily living) score pre-admission	0	13	3.3
	1	60	15.2
	2	34	8.6
	3	34	8.6
	4	49	12.4
	5	99	25.1
	6	105	26.6
	0–3 (dependent)	141	35.8
	4–6 (independent)	253	64.2
Discharge destination	Home	28	7.1
	Retirement home	94	23.9
	Healthcare residences	225	57.1
	Rehabilitation facilities	26	6.6
	Other intra-hospital departments	12	3
	Exitus	4	1
	Unknown	5	1.3

	Mean	SD	Range
Age	89.83	3.678	85–107
Total hospital stay (days)	13.54	5.909	3–40
Post-op hospital stay (days)	11.45	5.425	0–38

Statistical analysis of retrieved data for the whole population is reported in Tables 3 and 4.

**Table 3:** Association between mortality (intra-hospital, 30 days, 1 year) and timing of surgery (24, 48, 72 h) in the whole population

Mortality rate	Timing of surgical intervention		
	< 24 h (tot 230)	> 24 h (tot 711)	Chi-square
Intra-hospital	3 (1.3%)	29 (4.1%)	0.044
30 days	7 (3%)	35 (4.9%)	0.23
1 year	80 (34.8%)	212 (29.8%)	0.157

	< 48 h (tot 513)	> 48 h (tot 428)	Chi-square
Intra-hospital	7 (1.4%)	25 (5.8%)	< 0.001
30 days	15 (2.9%)	27 (6.3%)	0.012
1 year	138 (30.0%)	154 (32.2%)	0.463

	< 72 h (tot 622)	> 72 h (tot 319)	Chi-square
Intra-hospital	12 (1.9%)	20 (6.3%)	0.001
30 days	20 (3.2%)	22 (6.9%)	0.01
1 year	186 (29.9%)	106 (33.2%)	0.297

**Table 4:** Logistic regression between mortality (intra-hospital, 30 days, 1 year) and independent variables in the whole population

Variable	Parameter	End-points	*p*-Value	Odds ratio	95% CI
Gender	F	Intra-H	0.001	0.303	0.146–0.626
		30 days	< 0.001	0.299	0.158–0.568
	M	1 year	< 0.001	0.351	0.250–0.494
Fracture pattern	Medial	Intra-H	0.173	1.799	0.773–4.188
		30 days	0.023	2.443	1.134–5.266
	Lateral	1 year	0.168	1.240	0.913–1.686
Surgical technique	Fixation	Intra-H	0.277	0.669	0.324–1.328
		30 days	0.053	0.526	0.275–1.007
	Hip replacement	1 year	0.152	0.813	0.612–1.079
Type of anesthesia	General	Intra-H	0.083	2.401	0.893–6.458
		30 days	0.006	3.144	1.393–7.094
	Spinal	1 year	0.283	1.318	0.796–2.183
Timing of surgical intervention	≤ 24 h	Intra-H	0.056	0.311	0.094–1.030
		30 days	0.235	0.606	0.266–1.384
		1 year	0.158	1.255	0.916–1.721
Timing of surgical intervention	≤ 48 h	Intra-H	0.001	0.223	0.095–0.521
		30 days	0.014	0.447	0.235–0.852
		1 year	0.463	0.901	0.683–1.189
Timing of surgical intervention	≤ 72 h	Intra-H	0.001	0.294	0.142–0.610
		30 days	0.011	0.449	0.241–0.835
		1 year	0.297	0.857	0.642–1.145
Age (years)		Intra-H	0.393	1.042	0.948–1.147
		30 days	0.059	1.081	0.997–1.171
		1 year	< 0.001	1.091	1.049–1.135
Total hospital stay (days)		Intra-H	0.001	1.034	1.013–1.055
		30 days	0.100	0.963	0.920–1.007
		1 year	0.021	1.016	1.002–1.030

Early surgery within 48 and 72 h was significantly associated with a lower intra-hospital (*p* 0.001; OR 0.223; CI 0.095–0.521 and *p* 0.012 OR 0.294; CI 0.142–0.610*,* respectively) and 30-day mortality rate (*p* 0.014 OR 0.447; CI 0.235–0.852 and *p* 0.011 OR 0.449; CI 0.241–0.835, respectively). The possible correlation between surgery within 24 h and intra-hospital mortality (*p* 0.044) was not confirmed at the univariate analysis (*p* 0.056). No significant association was found for surgery within 24 h and 30-day mortality, as well as for early surgery (within 24, 48 or 72 h) and 1-year mortality rate (Table 3).

At univariate analysis, a significant association with a lower intra-hospital mortality rate was found for female sex (*p* 0.001; OR 0.303; CI 0.146–0.626). Conversely, length of hospital stay was associated with a significantly higher intra-hospital mortality rate (*p* 0.001; OR 1.034; CI 1.013–1.055). Regarding mortality at 30 days, a significant association with a lower mortality rate was found for female sex (*p* < 0.001; OR 0.299; CI 0.158–0.565), instead an association with higher mortality rate was found for general anesthesia (*p* 0.006; OR 3.144; CI 1.393–7.094) and intracapsular fracture pattern (*p* 0.023; OR 2.443; CI 1.134–5.266). Finally, a significant association with a lower 1-year mortality rate was found for female sex (*p* < 0.001; OR 0.351; CI 0.250–0.494), while age (*p* < 0.001; OR 1.091; CI 1.049–1.135) and length of hospital stay (*p* 0.021 OR 1.016; CI 1.002–1.030) resulted to be associated with a significantly higher 1-year mortality rate (Table 4).

Statistical analysis of retrieved data for the orthogeriatric subgroup is reported in Table 5.

**Table 5:** Logistic regression between mortality (intra-hospital, 30 days, 1 year) and independent variables in the orthogeriatric subgroup

Variable	Parameter	End-points	*p*-Value	Odds ratio	95% CI
Number of drugs per day		Intra-H	0.340	1.150	0.863–1.532
		30 days	0.402	1.084	0.898–1.308
		1 year	0.408	1.034	0.955–1.119
Anticoagulant	35	Intra-H	0.057	5.379	0.949–30.475
		30 days	0.137	2.711	0.727–10.107
	359	1 year	0.162	1.654	0.817–3.350
Antiaggregant	161	Intra-H	0.254	0.285	0.033–2.463
		30 days	0.945	0.963	0.336–2.762
	233	1 year	0.809	1.054	0.687–1.619
Antidiabetics	62	Intra-H	0.950	1.072	0.123–9.337
		30 days	0.344	0.372	0.048–2.884
	332	1 year	0.551	1.189	0.673–2.101
Acting on the CNS	197	Intra-H	0.138	5.104	0.591–44.093
		30 days	0.792	1.149	0.408–3.232
	197	1 year	0.451	0.850	0.557–1.297
Corticosteroids/immune-suppressive	18	Intra-H	0.190	4.365	0.483–39.446
		30 days	0.694	1.521	0.189–12.251
	376	1 year	0.919	1.054	0.386–2.875
Antiresorptive drugs	77	Intra-H	0.997	0.000	0.000
		30 days	0.997	0.000	0.000
	317	1 year	< 0.001	0.223	0.107–0.464
Comorbidities		Intra-H	0.057	1.872	0.981–3.572
		30 days	0.024	1.603	1.065–2.414
		1 year	0.001	1.361	1.143–1.620
Non-complicated hypertension	252	Intra-H	0.341	2.854	0.330–24.675
		30 days	0.199	2.317	0.643–8.351
	142	1 year	0.196	1.345	0.859–2.105
Cardiovascular (excluding non-complicated hypertension)	173	Intra-H	0.275	2.592	0.469–14.318
		30 days	0.080	2.650	0.889–7.903
	221	1 year	0.001	2.051	1.336–3.148
Respiratory diseases	45	Intra-H	0.115	4.012	0.714–22.554
		30 days	0.070	2.998	0.913–9.848
	349	1 year	0.238	1.468	0.776–2.778
Neurologic diseases(excluding dementia)	42	Intra-H	0.635	1.693	0.193–14.844
		30 days	0.733	1.304	0.284–5.987
	352	1 year	0.377	0.722	0.350–1.487
Metabolic diseases including diabetes	63	Intra-H	0.964	1.052	0.121–9.156
		30 days	0.335	0.365	0.047–2.828
	331	1 year	0.619	1.155	0.655–2.037
Renal failure	84	Intra-H	0.107	3.790	0.751–19.131
		30 days	0.021	3.432	1.207–9.756
	310	1 year	< 0.001	2.822	1.719–4.632
ADL (activities of daily living) score pre-admission		Intra-H	0.575	0.891	0.594–1.355
		30 days	0.407	0.896	0.691–1.162
		1 year	0.064	0.902	0.808–1.006
Dependent for ADL (score 0–3)	141	Intra-H	0.470	1.812	0.361–9.097
		30 days	0.157	2.114	0.750–5.957
Independent for ADL (score 4–6)	253	1 year	0.010	1.774	1.149–2.740
Post-operative delirium	57	Intra-H	0.997	0.000	0.000
		30 days	0.997	0.000	0.000
	337	1 year	0.103	0.578	0.299–1.117
*Discharge destination*					
Home	28	Intra-H	1.000	1.000	0.000
		30 days	0.529	2.046	0.221–18.982
		1 year	0.554	1.283	0.563–2.923
Retirement home	94	Intra-H	1.000	1.000	0.000
		30 days	0.438	1.821	0.400–8.301
		1 year	0.503	1.192	0.714–1.990
Other intra-hospital departments	12	Intra-H	0.994	> 1000	0.000
		30 days	0.009	11.050	1.805–67.642
		1 year	0.015	4.618	1.345–15.853
Rehabilitation facilities	26	Intra-H	0.995	> 1000	0.000
		30 days	0.486	2.210	0.238–20.553
		1 year	0.123	0.420	0.139–1.265
Healthcare residences	225	Intra-H	0.812	–	–
		30 days	0.147	–	–
		1 year	0.053	–	–

At univariate analysis, no parameter has proved to have a significant association with intra-hospital mortality. Conversely, a significant association with a higher 30-day mortality rate was found for transfer to other departments (*p* 0.009; OR11.050; CI 1.805–67.642), number of comorbidities (*p* 0.024; OR 1.603; CI 1.065–2.414) and renal failure (*p* 0.021; OR 3.432; CI 1.207–9.756).

Finally, a significantly lower 1-year mortality rate was found for antiresorptive drugs *(p* < 0.001; OR 0.223; 0.107–0.464), while a significantly higher 1-year mortality rate was found for transfer to other departments (*p* 0.015; OR 4.618; CI 1.345–15.853), number of comorbidities (*p* 0.001; OR 1.361; CI 1.143–1.620), cardiovascular diseases (*p* 0.001; OR 2.051; CI 1.336–3.148), renal failure (*p* < 0.001; OR 2.822; CI 1,719–4,632) and dependency in ADL (*p* 0.010; OR 1.774; CI 1.149–2.740).

A significant correlation between early surgery (48/72 h) and lower number of comorbidities (2 or less) was also found (*p* 0.002; OR 0.425; CI 0.250–0.723 and *p* 0.008 OR 0.431; CI 0.231–0.803, respectively). At multivariate analysis, no parameters were found to be statistically significant.

## Discussion

Early surgery for PFF is a highly debated topic. The rationale of early surgery for PFF resides in expected lower complications rate, reduced pain, faster rehabilitation and shorter hospitalization. The main goal however is recognized to be the reduction of mortality rate. Most studies set the goal of early surgery within 48 h from admission, supported by highly significant associations with lower mortality rate at different end-points [2, 4, 8, 19, 23, 24, 31–34, 38, 39]. However, some studies identify the threshold at 24 h to be significantly related to better outcome, both at short and mid-term [4, 19–30, 40]. Conversely, some studies set 72 h as the limit for early surgery, with more conflicting results [8, 24, 33]. Nonetheless, the goal of early surgery might be particularly difficult to reach in elderly patients, especially aged over 85 years, who are often frail and affected by several comorbidities. Indeed, some literature studies were not able to find benefits in terms of mortality rate in early surgical intervention [7, 34–37]. These authors underline that unstable and frail patients must be stabilized and properly prepared before intervention, even at the expense of operative timing [6, 7, 41]. Therefore, whether every patient beyond age, comorbidities and clinical conditions at admittance may benefit from this approach, is especially controversial.

Most studies that relate early surgery to mortality rate in PFF include patients aged over-65 [1, 2, 24], or in some cases over-70/75 [8, 19, 39]. However, the percentage of geriatric patients over-85 in PFF populations is generally growing. This group of patients, despite being representative of a small percentage of the whole population, accounts for nearly half PFF in many studies. In the present study, over-85 patients represented 46.5% of the whole PFF population, which is consistent with other literature studies reporting 42–50% of cases being over-85 [3, 4].

Including younger patients might lower the percentage of complex cases, making the goal of early surgery easier to reach. Moreover, these studies might include more active patients, compliant to rehabilitation and willing to return to pre-trauma level of activity. In the present paper focusing on over-85 patients, surgery was carried out within 48 h in 54.5% of patients, raising to 66.1% when considering the 72 h threshold. Conversely, only 24.4% of interventions was performed within 24 h. These data are consistent with other literature studies reporting 50–67% of interventions performed within 48 h [6, 7]. However, the percentage of early surgeries raised dramatically in the last years, as demonstrated in the more recent orthogeriatric subgroup data, where surgery was performed within 24, 48 and 72 h in 42.6%, 80.7% and 87.5% of cases, respectively. In the authors experience, the creation of an orthogeriatric service was determinant to reach this goal. This is also consistent with other more recent studies [7, 42–46].

Despite the efforts to optimize patients medical and surgical assistance, mortality rate remained high in this age group throughout the study period. In particular, 1-year mortality for the whole population was 31%, which is in line with the literature [1, 11–18]. Nonetheless, advanced age is clearly recognized as a risk factor for mortality in most studies [2, 7, 17, 19, 34, 47–49]. In the present study, even if focusing on an already advanced age group, overaged patients demonstrated a significantly higher mortality rate at 1 year. Moreover, 1-year mortality was not affected by early surgery in the present study, whatever the threshold considered.

Conversely, surgery within 48 and also 72 h was significantly related to lower intra-hospital and 30-day mortality rate in the present study. Correlation between surgery within 24 h and lower intra-hospital and 30-day mortality rate, although close to significance (*p* 0.056), was not confirmed at univariate analysis. This might be due to sample dimensions and deserves further investigations. Nonetheless, some recent literature reports question the value of the 24-h threshold for early surgery in geriatric patients [55–57]. First of all, as already suggested by some authors, benefits of early surgery in advanced age might not be as relevant as in younger patients [7, 23, 28, 34, 50]. Beside the already stated characteristics of elderly patients, the number of comorbidities might be a relevant issue to explain these findings. Complex patients with many comorbidities might develop post-operative complications despite early surgery [19, 47, 48, 51]. Likely, these patients might need time and clinical effort to reach acceptable pre-operative conditions [6, 7, 48, 52, 53]. One study in particular suggests that waiting more than two days for hip fracture surgery was not associated with higher complications or mortality rate if delay was due to stabilize patients with active comorbidities at admission [41]. In the present study, most patients had a relevant number of comorbidities, with very few having one or none. Despite studies are difficult to compare because of heterogeneous methods to categorize comorbidities and age cut-offs, these data compare negatively with other literature reports [1, 6, 8, 9, 17]. Moreover, the number of comorbidities in the present study were significantly associated to a higher mortality rate at 1 year. Likely, cardiovascular diseases and renal failure, which constitute the more common comorbidities in the study population, were both significantly associated with 1-year mortality. Some literature studies already recognized these associations [6, 46, 49, 54]. However, the reduced intra-hospital and 30-day mortality might be relevant enough to maintain the effort to perform surgery for PFF within 48 h also for advanced age patients. Moreover, differently from other studies [8], intra-hospital and 30-day mortality rates were significantly reduced also for surgery within 72 h. Despite there is general evidence against this delay in general PFF populations [22, 48, 53], it might be considered an acceptable goal in elderly patients with severe comorbidities that require some more corrective medical interventions to safely face the surgical procedure [23, 28]. Moreover, timing in the present study was significantly affected by number of comorbidities. Compromised patients (3 or more comorbidities) had surgery after 48 and 72 h in a significantly higher percentage of cases. Despite multivariate analysis could not identify the independent role of timing and comorbidities in influencing mortality, the present study results question timing to be the main variable in influencing mortality.

Nonetheless, when analyzing risk factors for mortality after PFF in this overaged population, number and type of comorbidities resulted to have a significant correlation with mortality, together with other parameters clearly recognized by the literature as risk factors as age and male sex [7, 17, 19, 34, 47, 48, 58]. Longer hospitalization, as well as transfer to other departments, showed a significant association with mortality at different end-points. These findings are not always recognized in the literature and may reflect the higher complexity of overaged patients. Surprisingly, a recent study by Nordström et al. reported that longer hospitalization seems to reduce early mortality rate [59]. Likely, being dependent in ADL at admission was associated to a higher 1-year mortality rate, which is consistent to some other recent studies [38, 39, 54, 60–64]. General anesthesia was associated to a higher 30-day mortality rate. This finding is not new, as many previous studies showed this association [65–67], while two recent meta-analysis reported contrasting results. Guay et al. did not show any difference between the two techniques, while Van Waesberghe et al. found a reduced intra-hospital mortality with spinal anesthesia but no difference at 30 days [68, 69]. However, whether type of anesthesia directly or indirectly affects mortality is still matter of debate [34], although its usefulness in controlling perioperative pain is well known [67]. In the literature, there are conflicting results about mortality according to fracture patterns. Gundel et al. and Holt et al. reported a higher mortality for extracapsular fractures, but they included in the study patients older than 18 and 50 years, respectively [34, 70]. Cornwall et al. also considered patients older than 50 years, but they divided hip fractures in four subgroups (nondisplaced femoral neck fractures, displaced femoral neck fractures, stable intertrochanteric fractures and unstable intertrochanteric fractures) and showed that displaced femoral neck fractures have the highest mortality rate [71]. We found a similar result, reporting that intracapsular fractures have globally higher mortality than extracapsular fractures, especially at 30 days.

Number and type of pharmacotherapies were not recognized as risk factors, except for antiresorptive drugs that showed a significant association with a lower 1-year mortality rate. This finding is consistent with some recent literature studies [58, 72, 73]. Nonetheless, it is not clear whether this result should be considered as a confounder, as stated by some authors, or if vitamin D and antiresorptive drugs may be directly related to lower mortality. Data of the present study do not allow to answer this statement.

Limits of the present study are: the retrospective design, the presence of detailed data for a limited part of the population, the single-center data collection, and the lack of a multivariate analysis because of the small amount of events (intra-hospital and 30-day death). Strengths of the study are the large sample and the thorough statistical analysis.

## Conclusions

In over-85 hip fracture patients, pre-operative stabilization and optimization of general conditions is mandatory together with surgery timing. In this age group, the threshold for early surgery might be moved to 72 h to allow medical optimization as intra-hospital and 30-day mortality rates remain significantly lower. Advanced age, male sex, number of comorbidities, pre-operative dependency in ADL, general anesthesia, length of hospitalization and transfer to other departments for medical conditions were all significantly related to mortality in over-85 patients. Number of comorbidities also significantly affected the timing of surgery. The possible protective role of antiresorptive drugs on mortality after PFF deserves more research and might be considered as confounding until different evidence.
